# A New Strategy for Fabricating Well-Distributed Polyaniline/Graphene Composite Fibers toward Flexible High-Performance Supercapacitors

**DOI:** 10.3390/nano12193297

**Published:** 2022-09-22

**Authors:** Yihan Qiu, Xiaoyu Jia, Mei Zhang, Hongwei Li

**Affiliations:** Beijing Key Laboratory of Clothing Materials R & D and Assessment, Beijing Engineering Research Center of Textile Nanofiber, School of Materials Design and Engineering, Beijing Institute of Fashion Technology, Beijing 100029, China

**Keywords:** polyaniline, graphene, composite fibers, energy density, fiber-shaped supercapacitors

## Abstract

Fiber-shaped supercapacitors are promising and attractive candidates as energy storage devices for flexible and wearable electric products. However, their low energy density (because their microstructure lacks homogeneity and they have few electroactive sites) restricts their development and application. In this study, well-distributed polyaniline/graphene composite fibers were successfully fabricated through a new strategy of self-assembly in solution combined with microfluidic techniques. The uniform assembly of polyaniline on graphene oxide sheets at the microscale in a water/N-methyl-2-pyrrolidone blended solvent was accompanied by the in situ reduction of graphene oxides to graphene nanosheets. The assembled fiber-shaped supercapacitors with gel-electrolyte exhibit excellent electrochemical performance, including a large specific areal capacitance of 541.2 mF cm^−2^, along with a high energy density of 61.9 µW h cm^−2^ at a power density of 294.1 µW cm^−2^. Additionally, they can power an electronic device and blue LED lights for several minutes. The enhanced electrochemical performance obtained is mainly attributed to the homogeneous architecture designed, with an increased number of electroactive sites and a synergistic effect between polyaniline and graphene sheets. This research provides an avenue for the synthesis of fiber-shaped electrochemically active electrodes and may promote the development of future wearable electronics.

## 1. Introduction

The ongoing development of portable and wearable electronics for health monitoring, implantable medical devices, intelligent robots and smart clothing has greatly improved our lives. Among various flexible energy storage and supply devices used for wearable electronics products, fiber-shaped supercapacitors with high flexibility and weavability have received a great deal of attention from both industrial and academic researchers [1,2,3,4]. Due to their typical properties of a light weight, large surface area, exceptional electric conductivity and good chemical stability, nanocarbon-based electroactive materials, such as graphene and carbon nanotubes, represent potential candidates as flexible electrode materials, especially graphene fibers [5,6,7]. However, pure graphene fibers display serious aggregation microstructures because of the strong π–π bonding in graphene sheets, resulting in them having a low specific surface area, poor electrolyte wettability and bad charge-storage ability. Therefore, a major challenge when we seek to develop practical applications for graphene fibers lies in solving their low energy density and achieving a stable energy output [8,9].

As we know, the electrochemical properties of a product are mainly dependent on the intrinsic properties of the electroactive materials. Most efforts made to improve the energy density and construct fiber-shaped supercapacitors with outstanding electrochemical performance have been devoted to three aspects: (a) Controlling the microstructure of graphene fibers. It is believed that ordered and porous pathways are beneficial for rapid ion diffusion and efficient charge transfer, as well as providing a large surface area to enhance the double-layer capacitance [10,11,12]; (b) The heteroatom doping (N, B, S, P) of graphene fibers. This is intended to tailor the surface polarity and electronic properties, thereby delivering extra pseudocapacitance to improve the energy density compared to that of pure graphene fibers [9,13,14,15]; (c) A homogeneous combination of graphene fibers with other electroactive materials, for example, transition metal oxides, metal sulfides and conducting polymers. This method is often utilized to mitigate the aggregation of graphene sheets in graphene fibers and improve electrochemical performance [16,17,18,19]. To achieve these objectives, a new strategy for developing microfluidic technology exhibits huge advantages for the architectural design, controllable compositions and organized functions of graphene-based fibers [20].

**Figure 1:**
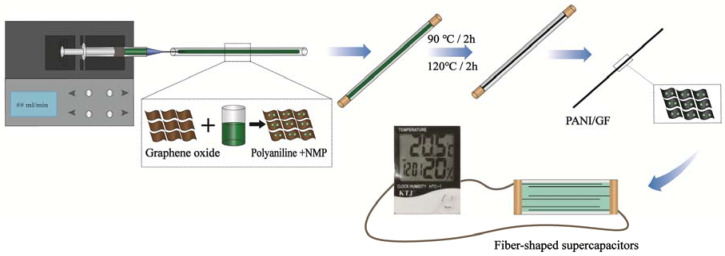
Schematic illustrations of the PANI/GF formation mechanism.

Polyaniline is a promising electrode material due to its high theoretical pseudocapacitance and electrical conductivity. This paper reports a novel preparation approach constituting molecular-scale self-assembly in solution combined with microfluidic techniques, to fabricate well-distributed polyaniline/graphene composite fibers (PANI/GF). The synthesis schemes are shown in Figure 1. The polyaniline uniformly anchored on graphene oxide sheets at the microscale in a water/N-methyl-2-pyrrolidone blended solvent is accompanied by the in situ reduction of graphene oxides to graphene nanosheets in a microreactor, which can provide more electroactive sites and guarantee a synergistic effect between polyaniline and graphene sheets to enhance the energy density and cycle stability. The fiber-shaped supercapacitors assembled by PANI/GF covered with PVA/H_2_SO_4_ and EMITFSI/PVDF-HFP gel-electrolyte demonstrate a large specific areal capacity of 541.2 mF cm^−^^2^ at 0.12 mA cm^−^^2^ and a high energy density of 61.9 µW h cm^−^^2^ at a power density of 294.1 µW cm^−^^2^, which is sufficient to power an electronic temperature and humidity meter and blue LED lights for several minutes. As such, graphene composite fibers possess advantageous characteristics for use in wearable energy-storage systems in prospective flexible/wearable electronic devices.

## 2. Materials and Methods

### 2.1. Reagents and Materials

All materials and chemicals were used without further purification. Aniline (C_6_H_7_N, 99.5%), ascorbic acid (C_6_H_8_O_6_, AR, 99.0%), N-methylpyrrolidone (NMP, AR, 98%), polyvinyl alcohol (PVA, AR, 99+%) and polyvinylidene chloride hexafluoryl (PVDF-HFP, average M_n_ ~130,000) were purchased from Aladdin Ltd., Shanghai, China. Ammonium persulfate ((NH_4_)_2_S₂O₈, AR, 98%) was purchased from Beijing Tongguang Fine Chemical Company, Beijing, China. Concentrated sulfuric acid (H_2_SO_4_, AR, 95–98%), hydrochloric acid (HCl, AR, 36–38%) and N,N-dimethylformamide (DMF, AR, 99.5%) were purchased from Jingchun Co., Ltd., Beijing, China, while 1-ethyl-3 methylimidazole bistrifluoromethyl sulfonimide salt (EMITSFI, 99%) was purchased from Shanghai Chengjie Chemical Co., Ltd., Shanghai, China.

### 2.2. Preparation of Polyaniline

Polyaniline (PANI) was synthesized by chemical polymerization. A total of 8 mmol of aniline monomer was added to 400 mL 1M hydrochloric acid solution and subjected to stirring for 5 min. Additionally, 8 mmol of ammonium peroxydisulfate was dissolved in 1M hydrochloric acid to obtain a uniform solution. The aniline solution was slowly poured into the ammonium peroxydisulfate solution (the molar ratio of aniline to ammonium peroxydisulfate was 1:1), accompanied by continuous stirring. The chemical polymerization reaction occurred at room temperature for 12 h. Then, polyaniline nanomaterials were obtained after filtration and cleaning processes with deionized water and ethanol, repeated several times, and dried in an oven at 60 °C.

### 2.3. Fabrication of Polyaniline/Graphene Composite Fibers

As shown in Figure 1, the fabrication process of polyaniline/graphene composite fibers (PANI/GF) was divided into two steps: firstly, graphene oxide (GO) solution was synthesized by a modified Hummers method, then sealed and stored at a low temperature. The GO solution was diluted to 5 mg mL^−1^ and placed in an ultrasonic bath for 1–2 h. After this, 10 mL GO solution and 0.033 g polyaniline dissolved in N-methyl-2-pyrrolidone (NMP) were distributed and mixed by ultrasound for 1 h. Then, 0.1 g ascorbic acid dissolved in deionized water was added to prepare the mixed spinning solution. Secondly, the spinning solution was injected into glass tubes using syringe pumps at a constant spinning rate. The sealed glass tubes were placed in an oven at 90 °C for 2 h, the gel fibers were preliminarily formed, and the temperature was increased to 120 °C for 2 h. After the fibers were completely formed, PANI/GF was dried naturally at room temperature. In the meantime, PANI/GF_1_ and GF were fabricated by the same method using 10 mL GO solution and either 0.033 g polyaniline dispersed in deionized water or no polyaniline.

### 2.4. Characterization

The morphology of the graphene oxides and polyaniline powder was characterized via transmission electron microscopy (TEM, JEOL JEM 2100, Tokyo, Japan). The morphology and EDX mappings of graphene-based fibers were measured by scanning electron microscopy (SEM, JEOL JSM-7600F, Tokyo, Japan). In addition, other information on the samples was produced through observations using XRD (Rigaku Smartlab SE, Tokyo, Japan) and X-ray photoelectron spectroscopy (XPS, ESCALAB 250, Waltham, MA, USA)

### 2.5. Preparation of Fiber-Shaped Supercapacitors and Electrochemical Measurements

The PVA/H_2_SO_4_ gel electrolyte was prepared as follows: 6 g PVA and 6 g concentrated H_2_SO_4_ were added to 60 mL deionized water under continuous stirring with 150 r.min^−1^, and the mixed solution was heated at 95 °C for 2 h until the suspension became clear.

The polymer-supported EMITFSI/PVDF-HFP gel electrolyte was successfully prepared. A total of 8 g ionic liquid of 1-ethyl-3-methylimidazolium bis(trifluoromethyllsulfonyl)imide (EMITFSI) and 12 g poly(vinylidenefluoride-co-hexafluoropropylene) (PVDF-HFP) were added to 60 mL dimethylformamide (DMF), and the solution was heated at 60 °C under continuous stirring until the mixture became clear.

Two parallel polyaniline/graphene composite fiber electrodes fixed on a flexible PDMS substrate were covered and wrapped with gel-like electrolyte to construct fiber-shaped flexible supercapacitors, and electrochemical measurements were taken after 1 h. An electrochemical workstation (CHI660E) was used to measure the electrochemical performance of CV, EIS and the galvanostatic charge/discharge cycling (GCD). The specific areal capacitance, energy density and power density are important indexes to evaluate the electrochemical properties of fiber-shaped supercapacitors. The specific areal capacitance (C, mF cm^−2^) of fiber-shaped supercapacitors can be obtained using the following formula:

$$C=\frac{4I\Delta t}{A\Delta V}$$

where I, ∆t, ∆V and A (cm^2^) are the discharge current (mA), discharge time (s), voltage range (V) and total surface area of two electrodes, respectively. The energy density (E) and power energy (P) are usually calculated with E = (CV^2^)/8 and P = E/∆t, where C (mF cm^−2^), V (V) and ∆t (s) are the specific areal capacitance, operating voltage and discharge time, respectively [21].

## 3. Results and Discussion

**Figure 2:**
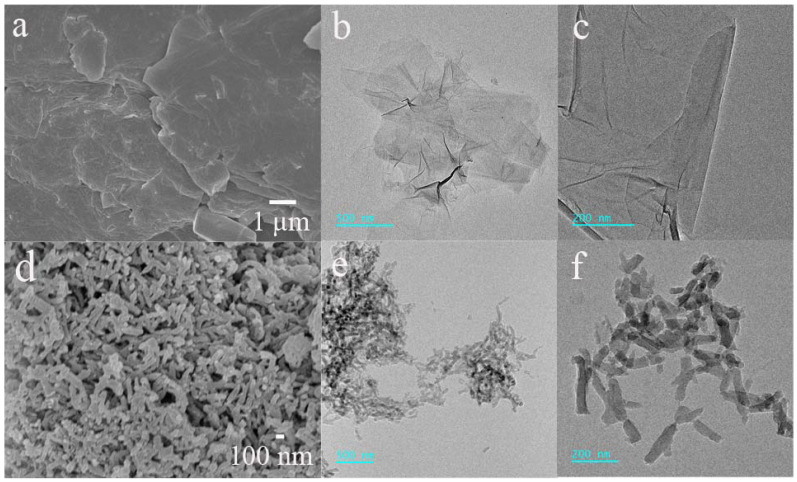
(**a**) SEM and (**b**,**c**) TEM images of GO, and (**d**) SEM and (**e**,**f**) TEM images of PANI.

The microstructures of graphene oxides (GO) and polyaniline (PANI) were characterized in detail using scanning electron microscopy (SEM) and transmission electron microscopy (TEM). Figure 2a–c show that the graphene oxides had a stacked and crumpled morphology caused by the van der Waals forces between the graphene oxide layers, revealing deformation because of the exfoliation and restacking processes that took place. As shown in Figure 2d–f, the polyaniline displays a nanofibrous structure with a diameter of about 50 nm and length extending from 100 to 200 nm. The uniform combination of fibrous polyaniline and graphene oxides cannot only effectively prevent the aggregation of graphene oxide layers and reduce the volume change of polyaniline but also increase the number of electrochemical active sites [22]. Then, we can obtain a homogeneous spinning solution of polyaniline/graphene oxides (PANI/GO) in a water/NMP blended solvent, indicating the good distribution of polyaniline on graphene oxide sheets (Figure S1a,b).

**Figure 3:**
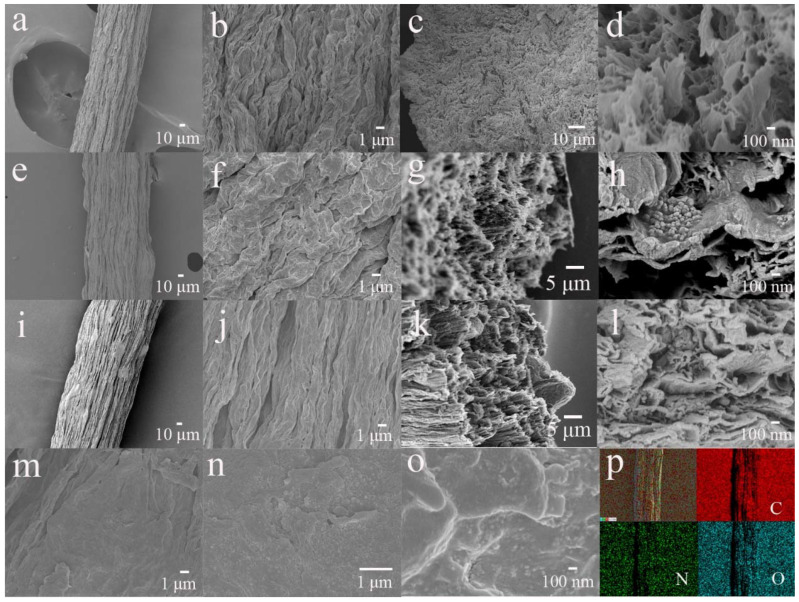
(**a**,**b**) Surface and (**c**,**d**) cross-sectional SEM images of GF; (**e**,**f**) surface and (**g**,**h**) cross-sectional SEM images of PANI/GF_1_; (**i**,**j**) surface and (**k**,**l**) cross-sectional SEM images of PANI/GF; (**m**–**o**) enlarged surface SEM images of PANI/GF; and (**p**) EDS mapping of C, O and N.

As shown in Figure 3, we used SEM to further investigate the structure and morphology of the fibers. GF, PANI/GF_1_ and PANI/GF indicated a diameter of approximately 155 μm and similarly rough surface with wrinkled and crumpled structures (Figure 3a,b,e,f,i,j). The cross-sectional morphology of GF presented a hierarchical and compact morphology with less porosity, few ionic transport channels and accessible surface areas (Figure 3c,d). PANI/GF_1_ and PANI/GF exhibited an obvious porous structure and different intercalation structure compared to GF, and polyaniline nanomaterials could be found on the surface and cross-section of PANI/GF_1_ (Figure 3g,h) and PANI/GF (Figure 3k,l). PANI/GF_1_ with uneven dispersion of polyaniline on graphene nanosheets may impede the electron transmission path, resulting in unstable electrochemical performance. During the preparation process of PANI/GF, the assembly of polyaniline on graphene oxide sheets at the microscale in a water/N-methyl-2-pyrrolidone (NMP) blended solvent is accompanied by the in situ reduction of graphene oxide to graphene nanosheets in a microreactor, which ensures molecular-level uniformity, and a strong interfacial interaction between polyaniline and graphene nanosheets, as shown in the enlarged surface SEM (Figure 3m–o) and TEM (Figure S1c,d) images. Energy-dispersive spectroscopy (EDS) mapping (Figure 3p) was utilized to characterize the spatial location distributions of the elements in PANI/GF. Visibly, the characteristic C, O and N elements can be seen in the composite fibers, and the well-distributed nitrogen elements belonging to polyaniline partly demonstrate the homogeneous loading of polyaniline on graphene nanosheets. Microfluidic-directed PANI/GF with a well-dispersed composition, well-developed porosity and good structural connectivity can considerably facilitate the high utilization of electroactive materials, allow for fast ions’ diffusion and enable the controllable electrochemical performance of fiber-shaped flexible supercapacitors.

**Figure 4:**
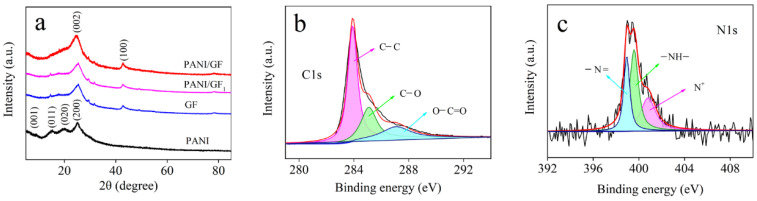
(**a**) XRD spectra of PANI, GF, PANI/GF_1_ and PANI/GF; (**b**) XPS C1s and (**c**) N1s spectra of PANI/GF.

As shown in Figure 4a, the XRD patterns of PANI, GF, PANI/GF_1_ and PANI/GF were analyzed. PANI had characteristic peaks at 9.2°, 14.7°, 20.4° and 25.2°, corresponding to the (001), (011), (020) and (200) crystal planes, respectively [23]. The (002) diffraction peak of GF at 25.5° indicated the disordered stacking of graphene sheets. The XRD patterns of PANI/GF_1_ and PANI/GF contained similar features to those of GF, both with a typical diffraction peak (002) around 25°. The intensity of this peak can be explained by the superposition effect between GF and PANI [24,25].

In addition, the high-resolution XPS of the C1s and N1s peaks was used to investigate the electronic states between elements. In Figure 4b, peaks at 283.9, 285.1 and 287.1 eV were assigned to the C-C in the aromatic rings, C-O of the epoxy and O=C-OH groups, respectively [26]. Compared to the GO spectrum shown in Figure S2, the oxygen content in PANI/GF was decreased significantly. The N1s in Figure 4c can be divided into three varying electronic states located at 398.9, 399.6 and 400.8 eV, corresponding to quinoid imine (−N=), benzenoid amine (-NH-) and the nitrogen cationic radical (N+), respectively [27]. The XPS results and the XRD spectra confirm the PANI structure in the composite fibers.

**Figure 5:**
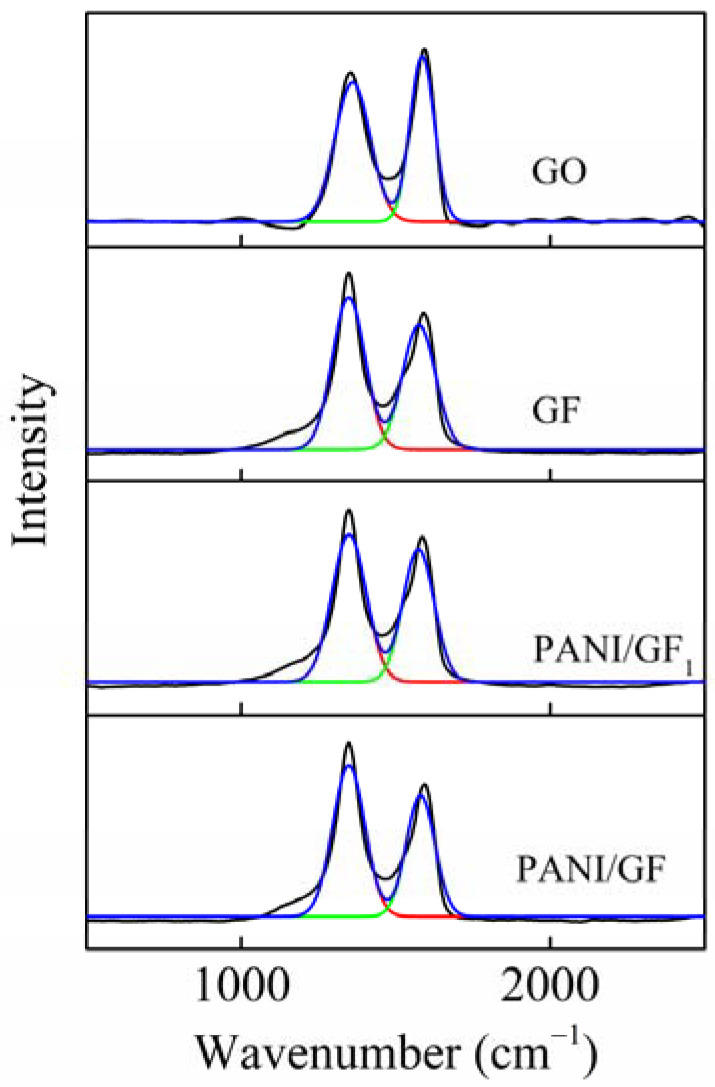
Raman spectra of GO, GF, PANI/GF_1_ and PANI/GF.

In Figure 5, the Raman spectra of GO, GF, PANI/GF_1_ and PANI/GF are shown. There were two typical peaks at 1353 and 1588 cm^−1^, attributed to the D and G bands, respectively, and no changes after the chemical polymerization reaction of polyaniline. PANI/GF indicated the highest I_D_/I_G_ value of 1.4 compared to GO, GF and PANI/GF_1_, demonstrating the introduction of more defect sites and the change in the grains’ size [25], which is beneficial for improving the electrochemical performance of flexible supercapacitors.

**Figure 6:**
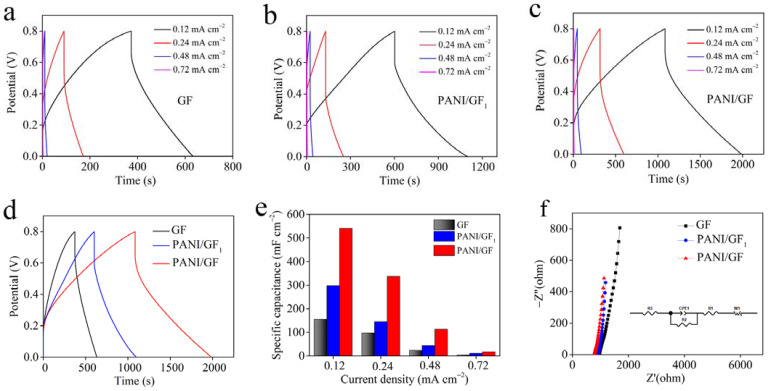
Electrochemical properties of GF, PANI/GF_1_ and PANI/GF in a two-electrode system measured using gel-state PVA/H_2_SO_4_ electrolyte. Galvanostatic charge-discharge (GCD) curves of (**a**) GF, (**b**) PANI/GF_1_ and (**c**) PANI/GF at various current densities of 0.12, 0.24, 0.48 and 0.72 mA cm^−2^; (**d**) GCD curves of GF, PANI /GF_1_ and PANI/GF at 0.12 mA cm^−2^; (**e**) specific areal capacitances of GF, PANI/GF_1_ and PANI/GF at various current densities, ranging from 0.12 to 0.72 mA cm^−2^; (**f**) EIS analysis of GF, PANI/GF_1_ and PANI/GF. Inset: equivalent circuit model.

Fiber-shaped flexible supercapacitors were assembled by the as-fabricated fibers coated with gel-state PVA/H_2_SO_4_ electrolyte to evaluate their electrochemical performance. Composite fibers with different amounts of polyaniline dissolved in NMP were synthesized and the electrochemical performance levels were obtained. The results are shown in Supplementary Material Table S1 and Figure S3. PANI/GF (PANI_0.033g_/GF) exhibited the longest charge–discharge times and the largest specific areal capacitance of 541.2 mF cm^−2^, greater than PANI_0.011g_/GF (213.0 mF cm^−2^), PANI_0.021g_/GF (342.6 mF cm^−2^) and PANI_0.050g_/GF (345.0 mF cm^−2^) at 0.12 mA cm^−2^. Figure 6a–e indicate the GCD curves and specific areal capacitances of GF, PANI/GF_1_ and PANI/GF at different current densities. According to the formula, PANI/GF prepared by polyaniline dissolved in NMP displayed specific areal capacitances of 541.2, 336.8, 112.8 and 17.3 mF cm^−2^ (for specific volumetric capacitances of 273.3, 170.1, 57.0 and 8.7 F cm^−3^) at the current densities of 0.12, 0.24, 0.48 and 0.72 mA cm^−2^, respectively. It is worth noting that the GCD curves of PANI/GF maintained nearly symmetric shapes without obvious redox peaks, suggesting rapid ions’ dynamic behavior and good reversibility [12,21]. When the current density was 0.12 mA cm^−2^, the specific areal capacitance of PANI/GF was higher than that of PANI/GF_1_ (298.2 mF cm^−2^) synthesized by polyaniline dispersed in water and that of pure graphene fiber (155.4 mF cm^−2^). The detailed specific areal capacitances calculated from the discharge curves of GF, PANI/GF_1_ and PANI/GF are shown in Figure 6e, indicating the best electrochemical performance of PANI/GF. In order to investigate the kinetic behavior of the electrolyte ions, the electrochemical impedance spectroscopy (EIS) spectra were measured (Figure 6f) and systematically analyzed. According to the equivalent circuit model, we can obtain the internal resistance, contact impedance of electrode materials, Warburg diffusion impedance and ion intercalation capacitance, respectively [21,28]. The detailed results analysis highlighted that the fiber-shaped supercapacitors exhibited the lowest impedance and Warburg diffusion impedance values, indicating their uniform microstructure and ions’ accessible electroactive surface were beneficial for ion transportation and accumulation [8,29,30,31,32].

**Figure 7:**
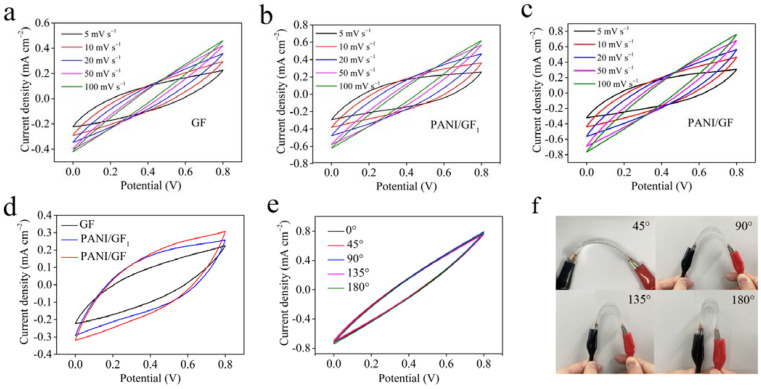
Cyclic voltammetry (CV) curves of (**a**) GF, (**b**) PANI/GF_1_ and (**c**) PANI/GF at different scan rates of 5, 10, 20, 50 and 100 mV s^−1^; (**d**) CV curves of GF, PANI/GF_1_ and PANI/GF at 5 mV s^−1^; (**e**) bending stability of PANI/GF under different angles at 100 mV s^−1^ and (**f**) photograph of bending tests.

Figure 7a–c show the cyclic voltammograms (CV) of the fiber-shaped flexible supercapacitors assembled using GF, PANI/GF_1_ and PANI/GF. There were operated in a narrow voltage window of 0–0.8 V at different scan rates from 5 mVs^−1^ to 100 mVs^−1^. The peak current densities of GF, PANI/GF_1_, and PANI/GF increased with the increasing scan rate. PANI/GF presented a wider current density range, indicating a rapid current response to the voltage changes and a good electrochemical performance [33]. The CV curve area reflected the charge-storage ability of the material. A comparison of GF, PANI/GF_1_ and PANI/GF is shown in Figure 7d. Compared to GF and PANI/GF_1_, the CV curve area of PANI/GF was larger, which proves that PANI/GF had the highest specific areal capacitance, high charge transfer and an excellent energy storage capacity [9,34,35]. It is significant that the fiber-shaped supercapacitors maintained their distinctive merits of excellent weavability and flexibility, broadening their range of practical applications in wearable electronic products [36]. To evaluate the bending deformation and stability of PANI/GF-based flexible supercapacitors, CV tests were performed at 100 mV s^−1^ under different bending angles of 0°, 45°, 90°, 135° and 180°, as shown in Figure 7e–f. It can be seen that the CV curves had no obvious changes at various bending angles, indicating the excellent bending stability and outstanding electrochemical performance of the material after multi-angle bending measurements [10,37]. In addition, we evaluated the long-term cycling stability, and the capacitance retention of PANI/GF was over 97% after 3050 cycles (Figures S4a and S5). A similar morphology and structure were found as for PANI/GF before the cycling test (Figure S4b). These results indicate good stability of PANI/GF-based wearable/flexible supercapacitors.

The enhancement of electrochemical performance was mainly achieved due to three factors, as follows: Firstly, polyaniline was dissolved in NMP during the synthesis process of PANI/GF, which facilitated the uniform loading of polyaniline onto graphene sheets in composite fibers to form an even morphology and guarantee structural stability. Secondly, the introduction of polyaniline with a high theoretical specific pseudocapacitance cannot only prohibit the restacking of graphene sheets to increase the number of electroactive sites available for energy storage but also improve the specific capacitance [26,38]. Finally, the uniform morphology of PANI/GF can improve the interfacial interaction between polyaniline and graphene sheets, enhance the contact between the electrode materials and electrolyte and accelerate the ion diffusion [28].

**Figure 8:**
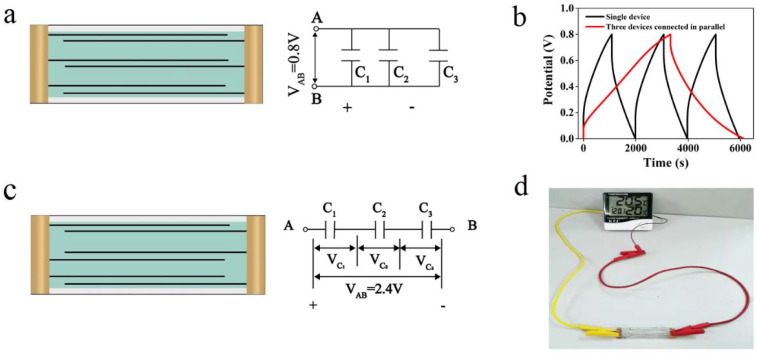
(**a**) Schematic illustration of three-fiber-shaped supercapacitors in parallel; (**b**) GCD curves of single- and three-fiber-shaped supercapacitors connected in parallel at a current density of 0.12 mA cm^−2^; (**c**) schematic illustration of three-fiber-shaped supercapacitors in series; (**d**) photograph of three-fiber-shaped supercapacitors in parallel used to power the electric thermo-hygrometer.

The investigation and development of fiber-shaped flexible supercapacitors aim to promote their application for smart clothes and portable electric devices. Multiple-fiber-shaped flexible supercapacitors are connected in parallel and in series to meet particular energy and power requirements [39]. The schematic illustration and GCD curves of three-fiber-shaped supercapacitors connected in parallel are shown in Figure 8a,b. The output current could also be enhanced by constructing three devices, and the discharge time was about three times that of a single-fiber-shaped supercapacitor at 0.12 mA cm^−2^ under the same operating voltage of 0–0.8 V [21]. In Figure 8c,d, it can be seen that by assembling three devices in series, the output voltage could be increased from 0.8 V to 2.4 V and the actual energy supply could power an electronic temperature and humidity meter for several minutes (Movie S1).

**Figure 9:**
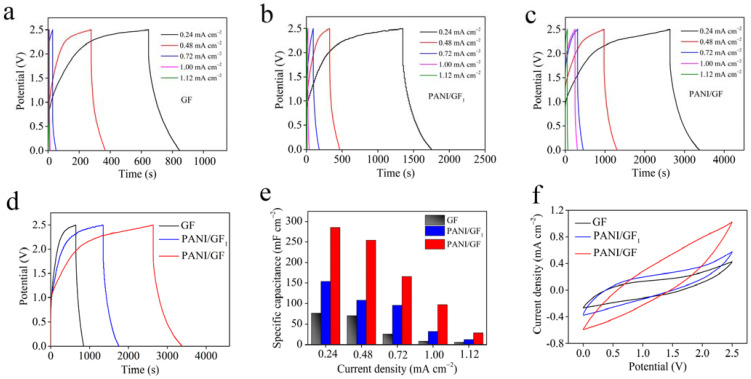
Electrochemical properties of GF, PANI/GF_1_ and PANI/GF in EMITFSI/PVDF-HFP gel-electrolyte. (**a**–**c**) GCD curves of GF, PANI/GF_1_ and PANI/GF at various current densities of 0.24, 0.48, 0.72, 1.00 and 1.12 mA cm^−2^; (**d**) GCD curves of GF, PANI /GF_1_ and PANI/GF at 0.24 mA cm^−2^; (**e**) specific areal capacitances of GF, PANI/GF_1_ and PANI/GF at 0.24, 0.48, 0.72, 1.00 and 1.12 mA cm^−2^; (**f**) CV curves of GF, PANI/GF_1_ and PANI/GF at a scan rate of 5 mV s^−1^.

The energy storage of flexible supercapacitors is proportional to the square of their operating voltage. Therefore, we utilized a polymer-supported ionic liquid (EMITFSI/PVDF–HFP) gel electrolyte, which has a wide electrochemical window and stability in air, to further evaluate the energy storage ability of the as-assembled supercapacitors, increase the energy density and broaden their potential application field [21,26]. As can be seen from Figure S6, the good reversibility was even more obvious with the voltage window reduced by between 1.5 V and 2.5 V, suggesting a manageable electrochemical window. The electrochemical performance levels are shown in Table S2. In Figure 9a–e, GCD curves of GF, PANI/GF_1_ and PANI/GF are illustrated at 0.24, 0.48, 0.72, 1.00 and 1.12 mA cm^−2^, as obtained by an electrochemical workstation. The GCD curves display a basically triangular shape with deviations from linearity and similar electrochemical behavior occurring under a wider potential window between 0 V and 2.5 V, revealing irreversibility to a certain extent, the same as in some previous literature [12,29,40,41]. The specific areal capacitance of PANI/GF was calculated to be 285.3 mF cm^−2^ (for a volumetric capacitance of 144.1 F cm^−3^) at a current density of 0.24 mA cm^−2^, which is larger than those of 153.6 mF cm^−2^ (specific volumetric capacitance of 77.6 F cm^−3^) calculated for PANI/GF1 and 76.8 mF cm^−2^ (specific volumetric capacitance of 38.8 F cm^−3^) calculated for GF. In addition, the flexible supercapacitor constructed using PANI/GF revealed a high areal energy density of 61.9 μW h cm^−2^ (volumetric energy density of 31.9 mW h cm^−3^) at a power density of 294.1 μW cm^−2^ (volumetric power density of 151.5 mW cm^−3^), which is superior to those previously reported for most graphene fiber-based flexible supercapacitors [1,21,26,31,34,42,43,44,45,46,47,48]. The detailed comparison results obtained for the electrochemical performance of graphene fiber-based flexible supercapacitors are shown in Table S3. Figure 9f shows the CV curves of the flexible supercapacitors assembled using GF, PANI/GF_1_ and PANI/GF at a scan rate of 5 mVs^−1^. PANI/GF presented a larger area than PANI/GF_1_ and featured pure graphene fibers under a wide voltage window from 0 V to 2.5 V. When constructing three devices connected in series, we found they had a high output voltage from 0 V to 7.5 V and could power blue LED lights (S5 S2). All the results obtained indicate that PANI/GF possesses excellent electrochemical properties, which can mainly be attributed to the significance of the well-distributed polyaniline in the composite fibers and the synergistic effect between the polyaniline and graphene sheets [16]. It was confirmed that the polyaniline/graphene composite fibers prepared by self-assembly in solution combined with microfluidic techniques exhibited great promise as flexible electrode materials for fiber-shaped supercapacitors, with the potential to accelerate the development of wearable energy storage devices and smart electronics.

## 4. Conclusions

In summary, in this research study, polyaniline/graphene composite fibers (PANI/GF) were fabricated through self-assembly in solution combined with microfluidic techniques. The as-designed architecture of polyaniline uniformly anchored on graphene sheets is beneficial for the improvement of energy density and cycle stability for flexible supercapacitors implanted in wearable electronic products. The assembled fiber-shaped supercapacitors have excellent electrochemical performance, for example, they have a specific areal capacitance of 541.2 mF cm^−2^ at 0.12 mA cm^−2^ and an energy density of 61.9 µW h cm^−2^ at a power density of 294.1 μW cm^−2^. These enhanced properties are mainly due to their molecular-level microstructure, their greater number of electroactive sites and the synergistic effect between polyaniline and graphene sheets. This current work reveals a potential strategy for precisely controlling the structure, morphology and composition of graphene-based fibers for flexible energy-storage devices, thereby broadening the practical applications for next-generation wearable electronics.

## Supplementary Materials

The following are available online at https://www.mdpi.com/article/10.3390/nano12193297/s1, Figure S1: TEM images of (a,b) PANI/GO solution in a water/N-methyl-2-pyrrolidone blended solvent and (c,d) PANI/GF powder dispersed in ethanol. Figure S2: XPS C1s spectrum of GO. Table S1: Polyaniline/graphene composite fibers with different amounts of polyaniline in NMP. Figure S3: GCD curves of PANI/GF with different amounts of polyaniline at a current density of 0.12 mA cm^−2^. Figure S4a: Cycling stability and capacitance retention at 0.35 mA cm^−2^; inset: GCD curves for the last ten cycles. Figure S4b: Surface (Ⅰ,Ⅱ) and cross-sectional (Ⅲ,Ⅳ) SEM images of PANI/GF. Figure S5: Cycling stability and capacitance retention at 0.8 mA cm^−2^; inset: GCD curves for the last ten cycles. Figure S6: GCD profiles of PANI/GF-based supercapacitors on EMITFSI/PVDF–HFP electrolyte at various operating voltages and a current density of 0.24 mA cm^−2^. Movies S1 and S2: Applications of fiber-shaped supercapacitors to power an electronic temperature and humidity meter (Movie S1) and blue LED lights (Movie S2) for several minutes. References [1,21,26,31,34,42,43,44,45,46,47,48] are cited in the Supplementary Materials.
