# Supplementary material for: A New Strategy for Fabricating Well-Distributed Polyaniline/Graphene Composite Fibers toward Flexible High-Performance Supercapacitors
Source: Nanomaterials (Basel). 2022 Sep 22;12(19):3297. doi: 10.3390/nano12193297 (PMC9565858; doi:10.3390/nano12193297)
Supplement: Supplementary file 1 [file nanomaterials-12-03297-s001.zip › nanomaterials-1887927-supplementary-done/Supplementary Material/nanomaterials-1887927-Supplementary Material-done.pdf]

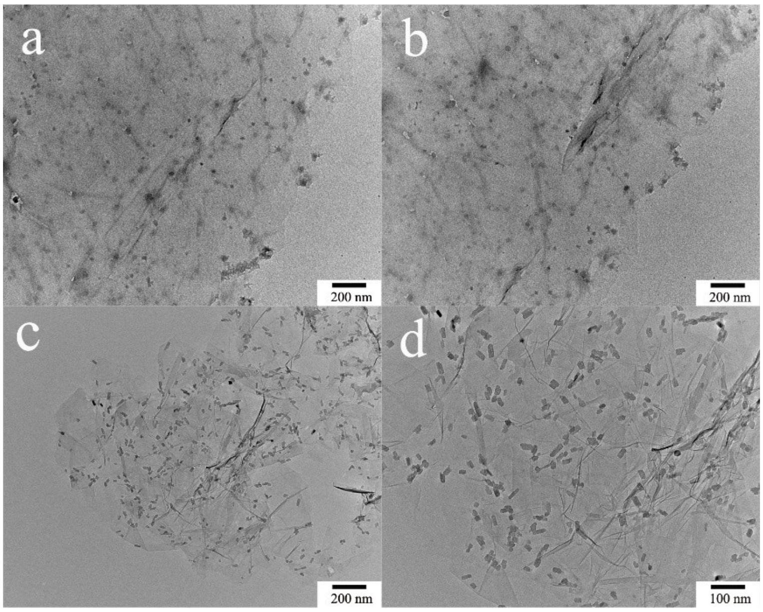

Figure S1. TEM images of (**a-b**) PANI/GO solution in a water/N-methyl-2-pyrrolidone blend solvent and (**c-d**) PANI/GF powder dispersed in ethanol.

PANI/GF were grinded into ultrafine powder and were distributed by using ethanol solution in an ultrasonic bath. Then we obtained the TEM images about PANI/GF.

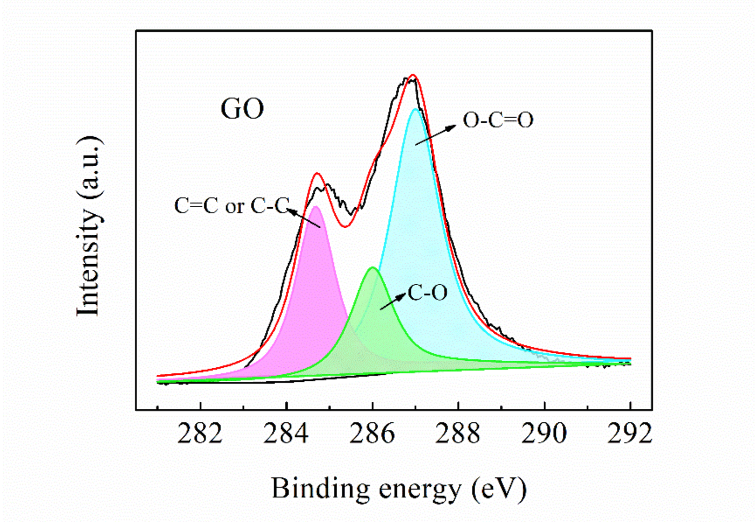

Figure S2. XPS C1s spectrum of GO.

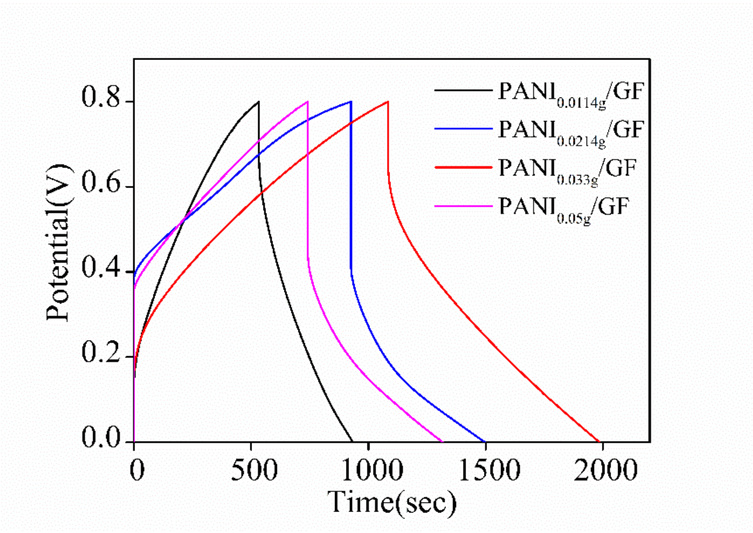

Figure S3. GCD curves of PANI/GF with different amount of polyaniline at a current density of 0.12 mA cm^{-2}.

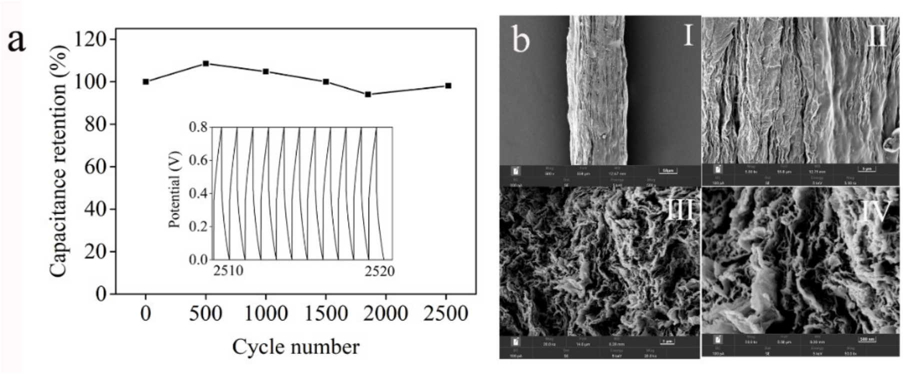

Figure S4. (a) Cycling stability and capacitance retention at 0.35 mA cm^{-2}, Inset: GCD curves for the last ten cycles. (b) surface (I-II) and cross-sectional (III-IV) SEM images of PANI/GF.

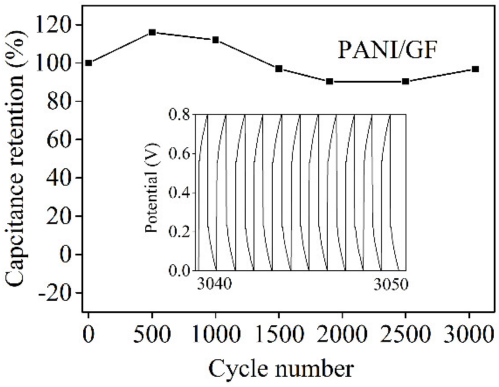

Figure S5. Cycling stability and capacitance retention at 0.8 mA cm^{-2}, Inset: GCD curves for the last ten cycles.

As shown in Figure S4-5, the capacitance retention of PANI/GF at 0.35 mA cm^{-2} is 98% after 2520 cycles, and the capacitance retention of PANI/GF at 0.8 mA cm^{-2} is 97% after 3050 cycles. The specific areal capacitance increases slightly during front portion of

charge/discharge processes, which may be ascribed to the redox reaction and volume change of polyaniline.

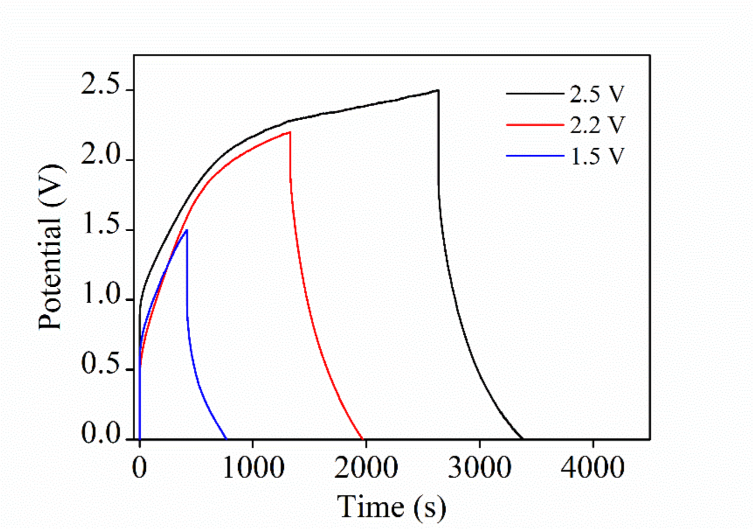

Figure S6. GCD profiles of PANI/GF based supercapacitors on EMITFSI/PVDF–HFP electrolyte at various operating voltages and current density of 0.24 mA cm^{-2}.

Table S1. Polyaniline/graphene composite fibers with different amount of polyaniline in NMP.

PANI/GF	Polyaniline (g)	Graphite oxides (g)	Areal capacitance (mF cm^{-2})
$PANI_{0.0114g}$/GF	0.0114 (18.6%)	0.05	213.0
$PANI_{0.0214g}$/GF	0.0214 (30%)	0.05	342.6
$PANI_{0.033g}$/GF	0.0330 (40%)	0.05	541.2
$PANI_{0.05g}$/GF	0.0500 (50%)	0.05	345.0

Table S2. Electrochemical properties of PANI/GF based flexible supercapacitors under various voltage window.

Voltage (V)	Charge time (s)	Discharge time (s)	C (mF cm^{-2})	E (μW h cm^{-2})
0-1.5 V	408	354	226.6	17.7

0-2.2 V	1328	638	278.4	46.8
0-2.5 V	2639	743	285.3	61.9

Table S3. Electrochemical properties of graphene fiber-based flexible supercapacitors.

Electrode Materials	Electrolyte	Specific Capacitance	Power Density	Energy Density	Ref.
GF or PANI/GF	PVA/H_3PO_4	3.3 or 66.6 mF cm^{-2}			[42]
GF	PVA/H_2SO_4	226 mF cm^{-3}	57.7 mW cm^{-3}	7.03 mW h cm^{-3}	[43]
GF	PVA/H_2SO_4 or PVDF/ $EMIMBF_4$	36.25 mF cm^{-2}	0.02 mW cm^{-2}	0.8 μW h cm^{-2} or 18.12 μW h cm^{-2}	[44]
RGO/PEDOT	PVA/H_3PO_4	304.5 mF cm^{-2}	66.5 μW cm^{-2}	27.1 μW h cm^{-2}	[1]
rGO/PEDOT	PVA/H_3PO_4	131 mF cm^{-2}		4.55 μWh·cm^{-2}	[45]
PPy/GF	PVA/H_2SO_4	147.9 F cm^{-3}	1300 mW cm^{-3}	13.15 mWh cm^{-3}	[46]
PPy/GF	PVA/H_2SO_4	107.2 mF cm^{-2}			[47]
PANI/GF	PVA/H_3PO_4 or EMITFSI/PVDF-HFP	230 mF cm^{-2}	15 mW cm^{-2}	37.2 μW h cm^{-2}	[21]
PANI/GF	PVA/H_2SO_4	357.1 mF cm^{-2}	0.23 mW cm^{-2}	7.93 μW h cm^{-2}	[31]
PANI/GF	PVA/H_3PO_4 or EMITFSI/PVDF-HFP	87.8 mF cm^{-2}	0.23 mW cm^{-2}	12.2 μW h cm^{-2}	[26]
PANI/GF	PVA/H_2SO_4	370.2 mF cm^{-2}	25.3 mW cm^{-2}	12.9 μW h cm^{-2}	[34]
PANI/GF	PVA/H_2SO_4	481 mF cm^{-2}	40.19 μW cm^{-2}	42.76 μW h cm^{-2}	[48]
PANI/GF	PVA/H_2SO_4 or EMITFSI/PVDF-HFP	541.2 mF cm^{-2}	294.1 μW cm^{-2}	61.9 μW h cm^{-2}	This work

1. Qu, G.; Cheng, J.; Li, X.; Yuan, D.; Chen, P.; Chen, X.; Wang, B.; Peng, H. A Fiber Supercapacitor with High Energy Density Based on Hollow Graphene/Conducting Polymer Fiber Electrode. *Adv. Mater.* **2016**, *28*, 3646–3652. https://doi.org/10.1002/adma.201600689.

21. Wu, X.; Wu, G.; Tan, P.; Cheng, H.; Hong, R.; Wang, F.; Chen, S. Construction of microfluidic-oriented polyaniline nanorod arrays/graphene composite fibers for application in wearable micro-supercapacitors. *J. Mater. Chem. A* **2018**, *6*, 8940–8946. https://doi.org/10.1039/c7ta11135e.

26. Zhang, M.; Wang, X.; Yang, T.; Zhang, P.; Wei, X.; Zhang, L.; Li, H. Polyaniline/graphene hybrid fibers as electrodes for flexible supercapacitors. *Synth. Met.* **2020**, *268*, 116484. https://doi.org/10.1016/j.synthmet.2020.116484.

31. Zheng, X.; Yao, L.; Qiu, Y.; Wang, S.; Zhang, K. Core–Sheath Porous Polyaniline Nanorods/Graphene Fiber-Shaped Supercapacitors with High Specific Capacitance and Rate Capability. *ACS Appl. Energy Mater.* **2019**, *2*, 4335–4344. https://doi.org/10.1021/acsaem.9b00558.

34. Yang, X.; Qiu, Y.; Zhang, M.; Zhang, L.; Li, H. Facile Fabrication of Polyaniline/Graphene Composite Fibers as Electrodes for Fiber-Shaped Supercapacitors. *Appl. Sci.* **2021**, *11*, 8690. https://doi.org/10.3390/app11188690.

42. Huang, T.; Zheng, B.; Kou, L.; Gopalsamy, K.; Xu, Z.; Gao, C.; Meng, Y.; Wei, Z. Flexible high performance wet-spun graphene fiber supercapacitors. *RSC Adv.* **2013**, *3*, 23957–23962. https://doi.org/10.1039/c3ra44935a.

43. Chen, S.; Ma, W.; Cheng, Y.; Weng, Z.; Sun, B.; Wang, L.; Chen, W.; Li, F.; Zhu, M.; Cheng, H.-M. Scalable non-liquid-crystal spinning of locally aligned graphene fibers for high-performance wearable supercapacitors. *Nano Energy* **2015**, *15*, 642–653. https://doi.org/10.1016/j.nanoen.2015.05.004.

44. Meng, J.; Nie, W.; Zhang, K.; Xu, F.; Ding, X.; Wang, S.; Qiu, Y. Enhancing Electrochemical Performance of Graphene Fiber-Based Supercapacitors by Plasma Treatment. *ACS Appl. Mater. Interfaces* **2018**, *10*, 13652–13659. https://doi.org/10.1021/acsami.8b04438.

45. Li, B.; Cheng, J.; Wang, Z.; Li, Y.; Ni, W.; Wang, B. Highly-wrinkled reduced graphene oxide-conductive polymer fibers for flexible fiber-shaped and interdigital-designed supercapacitors. *J. Power Sources* **2018**, *376*, 117–124. https://doi.org/10.1016/j.jpowsour.2017.11.076.
46. Liu, X.; Qian, T.; Xu, N.; Zhou, J.; Guo, J.; Yan, C. Preparation of on chip, flexible supercapacitor with high performance based on electrophoretic deposition of reduced graphene oxide/polypyrrole composites. *Carbon* **2015**, *92*, 348–353. https://doi.org/10.1016/j.carbon.2015.05.039.
47. Ding, X.; Zhao, Y.; Hu, C.; Hu, Y.; Dong, Z.; Chen, N.; Zhang, Z.; Qu, L. Spinning fabrication of graphene/polypyrrole composite fibers for all-solid-state, flexible fibriform supercapacitors. *J. Mater. Chem. A* **2014**, *2*, 12355–12360. https://doi.org/10.1039/c4ta01230e.
48. Wu, Y.; Meng, Z.; Yang, J.; Xue, Y. Flexible fiber-shaped supercapacitors based on graphene/polyaniline hybrid fibers with high energy density and capacitance. *Nanotechnology* **2021**, *32*, 295401. https://doi.org/10.1088/1361-6528/abf5fe.
